# Human microbiota, an emerging player in chronic obstructive pulmonary disease

**DOI:** 10.1016/j.ebiom.2026.106479

**Published:** 2026-09-09

**Authors:** eBioMedicine

According to a 2026 Global Burden of Diseases, Injuries, and Risk Factors analysis on chronic respiratory diseases, more than 200 million people were living with chronic obstructive pulmonary disease (COPD) worldwide in 2023, leading to a mortality burden of over 3 million people. An association between dysbiosis in the microbiome gut–lung axis and COPD severity and outcomes has been proposed and reviewed in the literature, although a direct causal link in humans remains to be determined.

The airway is exposed to the external environment and is home to a diversity of microbes. Researchers have asked whether the microbiome might play a role in the airway inflammation and remodelling that occurs with COPD. Based on 88 patients with COPD and 20 healthy control individuals, a study published in *Journal of Translational Medicine* in August, 2025, showed that COPD bronchoalveolar lavage fluid (BALF) samples were enriched with potentially pathogenic microorganisms (PPMs), such as *Streptococcus*, *Haemophilus*, and *Pseudomonas*, compared with the control individuals. Inter-individual variation and potential selection bias might limit the interpretation of the case–control studies. To address this problem, Meldrum and colleagues designed a longitudinal study to assess airway microbiome changes over time, published in the August, 2024, issue of *American Journal of Respiratory and Critical Care Medicine*. The authors prospectively assessed sputum samples and lung function in patients with COPD, and found a shift from commensal microbes, such as *Veillonella*, *Gemella*, *Rothia*, and *Prevotella*, to PPMs, including *Haemophilus*, *Moraxella* and *Pseudomonas*, when COPD progressed. Although the study does not provide a causal link, it suggests that targeting PPMs could provide a potential treatment pathway for COPD exacerbation.

In addition to airway microbes, gut microbiota might also contribute to the development of COPD. In a case–control study using 16S ribosomal RNA sequencing of human faecal samples, published in *Respiratory Research* in October, 2021, Li and colleagues showed a higher abundance of *Prevotellaceae* in the COPD GOLD severity stages 1 and 2 group, and a lower abundance of *Bacteroidaceae* and *Fusobacteriaceae* in the 3 and 4 group, compared with healthy control individuals. Viglino and colleagues further compared the microbiomes of 60 patients with stable COPD and 30 healthy controls through 16S rRNA sequencing. A comprehensive characterisation of oropharyngeal swabs, sputum, BALF, and stool samples was published in the January, 2026, issue of *eBioMedicine*. The authors found that oropharyngeal swab and BALF samples showed the highest number of differences for the taxa with differential abundance between patients and controls. 17 taxa were increased in BALF samples of the patients, including *Escherichia*, *Pseudomonas*, *Klebsiella*, *Staphylococcus* and *Akkermansia*, which is largely consistent with previous studies. However, most of the species associated with disease in the respiratory tract were not found in the stool samples, which showed differential abundance in only two taxa: *Klebsiella* and *Parabacteroides.* In addition, co-existing bacterial taxa in either airway or stool samples were associated with pertinent clinical traits, such as airflow limitation, exacerbation history, and inhaled steroid use. This study clearly shows the disparity of microbiome between patients and control individuals across different body compartments and suggests potential diverse contributions of human microbiota to the pathogenesis of COPD.

Although associative studies are informative, determining causal relationships requires in-depth mechanistic investigation. Microbial metabolites can regulate immune homoeostasis and modulate pulmonary inflammation, thus affecting COPD progression. Translational research published in the June, 2023, issue of *Cell Host & Microbe* showed that intranasal inoculation of *Staphylococcus aureus* significantly reduced lung function, increased tissue injury, and enhanced influx of neutrophils in an emphysema mouse model induced by lipopolysaccharide and elastase. Untargeted metabolomics revealed that homocysteine could be a crucial mediator, as inoculation of *metC* knockout *S aureus*, which produces substantially reduced amounts of homocysteine, resulted in attenuated lung function decline and less tissue damage, whereas homocysteine administration alone led to a similar phenotype as wild-type *S aureus*. A substantial upregulation of genes in neutrophilic degranulation, including S100A8, was observed in response to homocysteine treatment.

Another study published in the April, 2024, issue of *Gut* tested faecal microbiota transplant (FMT) in both directions between cigarette smoking-exposed and air-exposed mice. FMT from healthy, air-exposed mice into cigarette smoking-exposed mice reduced lung inflammation, marked by fewer leucocytes, neutrophils, and macrophages in BALF, and protected against alveolar destruction. In a separate experiment, FMT from cigarette smoking-exposed mice into air-exposed mice triggered lung inflammation, showing that cigarette smoking-altered gut microbiota can drive lung damage independent of direct exposure. Metabolomics and proteomics implicated downregulation of microbial glucose and starch metabolism in the development of COPD. A pilot randomised, double-blind, placebo-controlled trial was included to further explore the translational value of these findings. Patients with COPD took either inulin (n = 9), a complex carbohydrate that is hard for the host to digest but fuels the gut microbes, or placebo (n = 7) for four weeks. Fewer patients receiving inulin self-reported COPD exacerbations during the intervention period, compared with the placebo group (1 of 9 *vs* 4 of 7; p = 0·048). Although no differences in diversity of faecal microbiota in each individual were observed before and after inulin treatment, the abundance of *Bacteroides*, which usually process complex sugars, was significantly increased after inulin consumption (p = 0·01). Combined, these data suggest that microbial metabolites might have a direct role in mediating respiratory function, and diet supplements or FMT could be a potential therapeutic option for COPD.

Current research regarding the contribution of microbiota to COPD in humans is mainly associative and descriptive, whereas mechanistic exploration is largely performed in animal models. Translating preclinical findings into the human context is an ongoing challenge for the field. The rapid development of omics technologies, such as integrated gut metabolomics with host transcriptomics, will deepen our understanding of microbiome composition and their functions in mediating lung homoeostasis in humans. Meanwhile, large randomised controlled trials are necessary to test the therapeutic benefit of specific microbes and metabolites in the treatment of COPD. *eBioMedicine*, as a translational publishing platform, welcomes both preclinical and clinical research that helps inform our understanding of the complex relationship between the microbiome and human health.

